# Investigating appropriate molecular and chemical methods for ingredient identity testing of plant-based protein powder dietary supplements

**DOI:** 10.1038/s41598-019-48467-9

**Published:** 2019-08-20

**Authors:** Adam C. Faller, Thirugnanasambandam Arunachalam, Dhivya Shanmughanandhan, Prasad Kesanakurti, Hanan R. Shehata, Subramanyam Ragupathy, Steven G. Newmaster

**Affiliations:** NHP Research Alliance, College of Biological Sciences, University of Guelph, 50 Stone Rd E, Guelph, Ontario N1G 2W1 Canada

**Keywords:** PCR-based techniques, High-throughput screening, Protein sequencing, Mass spectrometry, DNA sequencing

## Abstract

Plant-based protein powders are rapidly growing in popularity, and outdated quality assurance tools expose vulnerabilities to adulteration via different methods of “protein spiking”. Adequate diagnostic tools are urgently needed to be able to authenticate protein source ingredients and screen for potential adulterants. We explored the application of three diagnostic tools for ingredient identification: targeted PCR with Sanger sequencing, NGS, and LC-MS/MS. We collected 33 samples of common commercial products from the plant-based protein powder market and sought to identify botanical components using the three technologies. We found success in detection with all approaches, with at least one main protein source being identified by at least one approach in all samples. The investigation uncovered challenges to data collection or result interpretation with each technology including but not limited to amplification biases with PCR technologies, potential influence of DNA degradation, and issues with protein solubility during isolation. Ultimately, each platform demonstrated utility along with certain caveats, which epitomized the importance of orthogonality of testing.

## Introduction

In a rapidly growing global dietary supplement industry (valued at USD $133.1 billion in 2016), development of regulatory policy must not be outpaced by emerging risks^1^. Dietary supplements may differ from pharmaceutical drugs in their exclusion from products able to claim ability to diagnose, cure, mitigate, or prevent a disease, but risk of unregulated, inauthentic or adulterated supplements is equally as dangerous and concerning to a consumer^2^. Supplements pass fewer regulatory checkpoints than their pharmaceutical drug counterparts before they become commercially available, which makes it all the more important that efficient, precise and accurate technology is used for quality testing^3^. In the United States, the dietary supplement industry has thrived, and the Food and Drug Administration’s (FDA) 1994 Dietary Supplement Health and Education Act is still the standard by which these products are defined and regulated^4^. Though constantly evolving, regulatory standards are struggling to efficiently address industry innovation, including new products or formulations. This has progressed to the point of the FDA commissioner recommending a complete overhaul of dietary supplement regulation^5^.

On the technical side of quality control, scientists must continue to develop new authentication protocols to contribute to comprehensive testing regimes for all different types of products. The amount of different dietary supplements on the market has increased by over twenty times, with over 90 000 sold in the United States in 2014^3^. Evaluating quality and identity testing options for this overwhelming figure calls for initiation of explorative studies designed to assess techniques as they apply to specific ingredients and products. Thus, we propose methodology such as that followed in this paper, where we compare multiple techniques — targeted PCR, Next Generation Sequencing (NGS), and Liquid Chromatography with tandem Mass Spectrometry (LC-MS/MS) — in the context of a case study. We chose to explore protein powders with a focus on the rapidly growing assortment of plant-based options.

The global movement to popularize plant-based diets for sustainability, economic or health reasons is consistently expanding, with many consumers seeking plant-based alternatives to satisfy their dietary needs^6^. When adopting a restrictive diet such as veganism or vegetarianism, it can be difficult to maintain a balanced nutrient intake. For this reason, many turn to meal supplementation with products such as protein powders. The global protein supplement market was valued at USD $12.4 billion in 2016 with a projected CAGR of 6.3% from 2017 to 2025^7^. Within this market, protein powders alone contributed to 64.5% of overall revenue in 2016^7^. Though animal-based proteins still command the largest portion of the supplement market, multiple sources predict plant-based proteins to experience the quickest growth, with CAGR projections reaching as high as 7.9%, leading to market valuations as high as USD $16.3 billion by 2026^7–9^.

In any growing market with supply racing to meet demand, there is a risk of economically motivated adulteration^10^. The plant-based protein powder market is clearly on a path of rapid growth, thus highlighting an imperative need for adequate quality assurance programs. Current protein quality testing is largely concerned with protein content measurement. Though outside the scope of ingredient identification, it is important to note that current methods to estimate protein content are outdated and indirect^11,12^. The Kjeldahl and combustion (Dumas) methods for measuring total protein content are based on measuring nitrogen content of a sample. These indirect methods leave products open for adulteration with any substance intended to artificially increase nitrogen content, as has already been identified in multiple cases^10^. Some of these events may be relatively harmless, like in the case of “spiking” powders with free amino acids, but others can be dangerous, such as the addition of compounds like melamine — a toxic compound used to boost nitrogen that has led to fatalities in some cases^13^. These instances of economically motivated adulteration reveal the current susceptibility of this market. Rapid growth only behooves industry to race to develop fit for purpose quality testing tools that address all possible sources of adulteration. From a safety standpoint, consumers should be able to trust ingredient labels on all protein powder products and make decisions based on their health goals and avoidance of potential allergic reactions. As with the rest of the natural health product (NHP) industry, premium botanical-based products can come with a substantial price tag, and consumers deserve to have confidence in quality and authenticity of products they purchase.

We have recently demonstrated the ability to extract DNA from processed plant-based protein powders and use that template DNA for ingredient identification via targeted PCR and sequencing (unpublished). This is consistent with demonstrated success in using DNA extracted from processed NHPs to identify botanical material. Protein powders go through various intensities of production, depending on what protein source is being used or what product type is being created (e.g., concentrates, isolates, hydrolysates etc.)^14^. Procedures are focused around concentrating the protein component of a starting organic material. As long as DNA of sufficient quantity and template integrity is retained through processing, molecular approaches can be applied to ingredient authentication. Better tools are urgently needed to address vulnerabilities in quality control programs that can be exploited for the purpose of adulteration.

The objective of this study is to evaluate the efficacy of different technologies applied to ingredient identification in protein powders, guided by three main lines of questioning: 1) Can targeted PCR, NGS and LC-MS/MS be used for protein ingredient identification in plant-based protein powders, and how do results compare? 2) Can powders be screened for whey presence and GMO-soy presence using hydrolysis probe based assays? 3) Can any unlisted ingredients be detected using PCR, NGS and LC-MS/MS, and how do results compare?

## Results

The purpose of this study was to investigate the ability of three technologies to identify ingredients within common plant-based protein powder products. We divided this focus into three main goals, including: (1) an attempt to identify main protein source ingredients listed on label, (2) a hydrolysis probe screen of all products for whey protein and GMO-soy presence, and (3) an attempt to detect possible adulterants. With a trifold ingredient screen using each method, results from any given technology could be validated against the other two.

### Identification of protein sources within products

Thirty-three protein powder samples featuring one to four main protein source ingredients were investigated using three approaches: targeted PCR with Sanger sequencing, NGS of the *ITS* or *rbcL* region, and LC-MS/MS amino acid sequencing. Results indicating success of detection for main protein sources are summarized in Table 1.

**Table 1:** Summary of detection success for main protein ingredient screen and probe screen.

Sample Code	Ingredients	Main Ingredient Detection			Probe Screen	
		PCR + Seq	NGS	LC-MS/MS	GMO Soy	Whey (*Bos taurus*)
P1	*Glycine max*	∙	∙	∙		
P2	*Glycine max*	∙	∙	∙		
P3	*Salvia hispanica*	∙	∙			
P4	*Cucurbita* sp.	∙	∙	∙		
P5	*Cannabis sativa*	∙	∙			
P6	*Cannabis sativa*	∙	∙			
P7	*Cannabis sativa*	∙	∙			
P8	*Cannabis sativa*	∙	∙			
P9	*Cannabis sativa*	∙	∙			
P10	*Cannabis sativa*	∙	∙			
P11	*Cucurbita* sp.	∙	∙	∙		
P12	*Cucurbita* sp.	∙	∙	∙		
P13	*Oryza sativa*	∙	∙	∙		
P14	*Oryza sativa*	∙	∙	∙		
P15	*Oryza sativa*	∙	∙	∙	∙	
P16	*Oryza sativa*	∙	∙	∙		
P17	*Oryza sativa*	∙	∙	∙		
P18	*Oryza sativa*	∙	∙	∙		
P19	*Oryza sativa*	∙	∙	∙		
	*Chenopodium quinoa*	∙	∙			
P20	*Pisum sativum*	∙	∙	∙		
P21	*Pisum sativum*	∙	∙	∙		
P22	*Pisum sativum*	∙	∙	∙		
P23	*Pisum sativum*	∙	∙	∙	∙	
P24	*Pisum sativum*	∙	∙	∙		
P25	*Pisum sativum*	∙	∙	∙	∙	
	*Oryza sativa*			∙		
	*Cannabis sativa*					
	*Chenopodium quinoa*					
P26	*Pisum sativum*	∙	∙	∙		
	*Cannabis sativa*	∙	∙			
	*Oryza sativa*	∙		∙		
	*Salvia hispanica*	∙	∙			
P27	*Pisum sativum*	∙	∙	∙		
	*Oryza sativa*			∙		
P28	*Oryza sativa*	∙		∙		
	*Pisum sativum*	∙		∙		
	*Cannabis sativa*					
P29	*Pisum sativum*	∙	∙	∙		
	*Cucurbita* sp.					
	*Oryza sativa*			∙		
P30	*Pisum sativum*	∙	∙	∙	∙	
	*Cannabis sativa*	∙				
	*Salvia hispanica*					
	*Chenopodium quinoa*					
P31	*Pisum sativum*	∙	∙	∙	∙	
	*Oryza sativa*			∙		
	*Cannabis sativa*	∙	∙			
P32	*Oryza sativa*			∙		
	*Pisum sativum*	∙	∙	∙		
P33	*Glycine max*	∙		∙	∙	

Note: “∙” refers to a positive detection.

We were able to detect at least one main protein source for each of the 33 samples using at least one of the detection methods. In 16 out of 33 samples, we were able to detect the main protein sources using all three methods. To note, those 16 samples each had only one main protein source. The full 33 samples included a total of 51 tested ingredients. Six out of 51 tested ingredients in a total of four samples were not detected using any of the three detection methods. These four samples (P25, P28, P29 & P30) included a mixture of protein sources.

With regards to the coverage of each method, of the 45 detected ingredients, targeted PCR with Sanger sequencing was able to detect a total of 40 ingredients (89%), NGS a total of 35 ingredients (78%), and LC-MS/MS a total of 33 ingredients (73%).

### Hydrolysis probe based real time PCR for GMO soy and whey protein

All 33 samples were screened for GMO-soy using a CP4 gene based probe, as well as for whey adulteration using a probe targeting *Bos taurus*. Six of the 33 samples showed positive signals for GMO-soy while none of the samples showed positive hits for whey (Table 1).

### Investigation of possible adulterants

Further to seeking confirmation of main protein source ingredients for each product, we explored a handful of common adulterants. Those included secondary ingredients listed on some of the product labels as well as a few unlisted species that researchers considered likely to be a protein boosting adulterant: *Zea mays* (maize)^15^, *Triticum aestivum* (wheat)^15^, *Vica faba* (fava bean)^16^, *Cicer arietinum* (chickpea)^17^, *Cyamopsis* sp. (guar or cluster bean)^18^ and *Medicago sativa* (alfalfa grass)^19^. The seven main protein sources explored in this study were also noted as adulterants if present in a product without being listed.

Using targeted PCR, we screened for the above-mentioned adulterants and detected one or two unlisted species in 15/33 products (Supplementary Table S1). NGS identified a variety of non-listed species, but all represented sub 1% of the read count (Supplementary Table S1). In the LC-MS/MS run that was preceded by a TRIzol® based protein isolation (before trypsin digestion), no adulterants were detected for any products; however, a host of potential adulterants for 25 products were revealed via the round of samples that did not undergo a protein isolation prior to trypsin digestion (Supplementary Table S1). Overall, two samples (P1 and P4) revealed an adulterant detected with all three methods, and seven samples revealed an adulterant detected using two of the three methods (Table 2).

**Table 2:** Summary of adulterant detection for botanicals detected by at least two techniques.

Samples	Adulterants	PCR	NGS	LC-MS/MS
P1	*Pisum sativum*	∙	∙	∙
P4	*Glycine max*	∙	∙	∙
P9	*Pisum sativum*		∙	∙
P12	*Glycine max*		∙	∙
P13	*Cucurbita* sp.	∙		∙
P17	*Pisum sativum*	∙		∙
P24	*Glycine max, Vicia faba*		∙	∙
P31	*Cicer arietinum*		∙	∙
P33	*Pisum sativum*	∙		∙

Note: “∙” refers to a positive detection

### Reference samples

Up to fourteen vouchered reference samples were tested alongside test samples in each technique to ensure detectability (Supplementary Table S2). All references were successfully identified using targeted PCR and NGS, and 11/14 were identified using LC-MS/MS. DNA was extracted and protein was isolated from the protein containing portion of the reference botanical sample. The samples not identified using LC-MS/MS included *Salvia hispanica*, *Cannabis sativa* and *Cyamopsis* sp.

## Discussion

Plant-based protein powders are an important example of a dietary supplement that is rapidly growing in popularity and has shown key vulnerabilities in quality and safety^20^. In order to design comprehensive quality assurance protocols, the efficacy of multiple approaches must be investigated as they pertain to different ingredients and products.

The first goal of this study was to evaluate the ability of the three technologies to detect presence of listed main protein sources. Positive results were supported via simultaneous testing of reference samples for each ingredient. DNA based approaches to botanical authentication have been accepted by regulatory bodies as a promising addition to quality assurance programs, worthy of validated method development^21^. Recent literature has importantly outlined the proper considerations in molecular assay methods development, discussing protocol considerations like DNA extraction strategy, genomic region selection, primer design, amplicon size, PCR optimization, and appropriate voucher reference libraries; this comprehensive approach ultimately leads to validated methods^21–26^. It is imperative that assay development adheres to universal analytical development guidelines, such as those of AOAC, in order to properly evaluate applicability and allow for reproducible global implementation^27^.

To date, there has not been any detailed investigation into the application of molecular authentication methods to protein powder supplements. Like many supplements, these products are heavily processed — in this case, for the goal of dietary protein isolation and concentration — and residual DNA within products may be degraded or lost^28^. However, this understanding should only be viewed as a caution in considering negative molecular detection results as true negatives. Past work has demonstrated that useful DNA (used for the purpose of identification) can remain in highly processed products^28^. Thus, DNA based techniques should be considered for all processed supplements. In this study, targeted PCR (with Sanger sequencing) was able to identify at least one ingredient in 100% of the samples, and NGS, 94%. Considering ingredients regardless of sample, in the 45 ingredients detected by at least one method, targeted PCR and NGS were able to detect 89% and 78%, respectively. Furthermore, both techniques were able to detect all different types of protein sources. This high success of detection demonstrates the utility of residual DNA for identification. There are plenty of examples in literature of ongoing development and application of DNA based identification applied to the protein source botanicals included in this study: soy, pea, rice, hemp, quinoa, chia, pumpkin^29–38^. Interestingly, in five cases in our study, NGS could not detect an ingredient where targeted PCR could. This exemplifies the trade-off between use of universal vs specific primers. While design of universal primers around a barcoding region may avoid time consuming development and optimization of multiple primer sets, species specific primer set development may lead to overall greater and more reliable success in detection.

All main protein ingredients’ presence was confirmed via at least one method, but interestingly, the main protein sources were able to be detected with all three methods in only half of the samples. This suggests the importance of considering method and protocol suitability for different ingredients. For example, we found that we were never able to identify *Cannabis sativa* or *Salvia hispanica* proteins in the product samples or the reference samples using LC-MS/MS. Regardless of sample, *Cannabis sativa* protein was never able to be detected using LC-MS/MS (in 11 instances) when comparatively it was detected by PCR with Sanger sequencing 82% of the time and NGS 73% of the time. A previous study by Tang *et al*. investigated the physiochemical and functional properties of the protein isolate from hempseed and determined that edestin, the main storage protein component of hemp seeds, had poor solubility in sub 7.0 pH solutions^39^. They suggested that a high cystine and methionine residue content within edestin encouraged covalent disulfide bond formation among molecules, especially at a low pH, leading to aggregation^40^. Additionally, a study conducted in 2012 by Park *et al*. sought to profile hempseed proteins via two-dimensional gel electrophoresis and LC-MS/MS; they also could not identify edestin^41^. They attempted to suspend a crude protein extract in a buffer and visualize it via 2D SDS-PAGE (8–16% gradient polyacrylamide gels) and identify proteins (excised from the gel and digested with trypsin) via LC-MS/MS. They cited reasons for unidentifiability as insolubility of edestin in the homogenation buffer (pH 7.5) used in their 2-DE protein extraction^41^. We encountered similar difficulty with *Salvia hispanica*. Previous studies investigating the chemical properties of chia seed proteins uncovered a strong effect of pH on solubility, revealing poor solubility of chia seed globulins at a low pH and better solubility in alkaline conditions^42^.

Nevertheless, LC-MS/MS appears to be a tool gaining popularity for the purpose of protein identification via amino acid sequencing. Validated methods are beginning to be published, even in the protein space with ESI HPLC-MS/MS protocols for pea, rice, soy, whey and casein proteins in raw materials and finished goods (AOAC 2017.11 and AOAC 2017.12)^43,44^. Successful application of these protocols to quality testing was recently published in an industry report by Dyad Labs^45^. They focussed on the five protein sources for which AOAC validated methods existed and were able to confirm the presence of the main source in 25 tested samples, using LC-MS/MS. The limited number of ingredients studied shows that there is still plenty of development required to validate protocols for all other main protein sources, namely those that are plant derived.

The secondary focus of this study was to evaluate application of molecular and chemical technologies to screen for potential adulterants. The hydrolysis probe assay for whey (*Bos taurus*) detection as well as the one for GMO soy detection both successfully rendered positive signals for reference samples. The *Bos taurus* probe did not detect whey presence in any of the 33 samples, suggesting absence of whey, or at least absence of *Bos taurus* DNA. This is a prime example of the importance of correctly interpreting molecular diagnostic results. Though presence of *Bos taurus* DNA should be viewed as a suggestion of whey presence (and indication to seek confirmation using another technology), a negative result may be due to absence of whey, or simply degraded DNA. Thus, results should be considered subject to possible false negatives when DNA presence is being used as a proxy for protein presence. GMO-soy presence was also investigated via hydrolysis probe, revealing six samples with positive hits (four with non-GMO product claims). Detailed product authentication was not the main focus of this study, as such investigations should be carried out with validated methods available for all tested ingredients. However, in an explorative capacity, some of this work’s results suggest possible avenues for further research and market surveys. GMO-soy presence in non-GMO soy claiming products is a violation of consumer trust, and falsely advertises a premium feature of what is a typically more expensive product. Ninety-four percent of the soy crop in the United States is GMO soy (and 80% of the global soy crop), thus adherence to non-GMO claims should be a fixture of authenticity testing in such products^46,47^.

In this study, we also used the three main technologies (PCR with Sanger sequencing, NGS and LC-MS/MS) to investigate adulterants. Interestingly, specific adulterants were detected by more than one method in only 9/33 samples, despite estimates of potential adulteration reaching as high as 76% with LC-MS/MS. Full lists are included in Supplementary Table S1.

The shortcoming of positive PCR or NGS results is that we are unable to distinguish positive signals as being the result of substantial presence of an organic adulterant, or that of incidental DNA due to minor contamination during production. Since its advent, next generation sequencing has captured great excitement in the scientific community as an attractive research tool, prime in its sensitivity, ability to generate reads from multiple DNA sources in one run, and wide variety of applications^48^. As demonstrated in this study, it can be applied to botanical detection in finished dietary supplements, though potential biases may complicate data interpretation. It is common for food and dietary supplements to include at least trace amounts of other botanical species that may have been inadvertently introduced to the supply or production chain at points such as raw material harvest if species were growing in the same location, or processing if different botanical products were produced in the same factory^49^. These trace contaminants are acknowledged in regulatory guidelines (low acceptable thresholds are sometimes outlined) and negligible amounts should not be considered true adulteration^49^. However, the sensitivity of NGS, coupled with potential inflation of read count due to primer bias, can easily lead to false positive tests for adulterants^49,50^. In fact, NGS is open to a wide variety of biases that can lead to false positives, false negatives and incorrect estimates of relative read quantity^51^. NGS is particularly sensitive to bias in the early steps of library preparation — an essential enrichment step in many pipelines that uses PCR to amplify target DNA starting template^52^. Differences in universal primer affinity, GC content of an amplicon, DNA secondary structure, and imprecise amplification at high cycle numbers (i.e., rehybridization of PCR products) can result in substantial differences in amplification efficiency^53–55^. Universal primers may be of such low affinity to some species’ priming sites that they don’t bind in any appreciable amount, resulting in false negatives. Conversely, they may be of an exceptionally high affinity to other species, whose sequences may outcompete others during annealing of primers, and thus will ultimately render misleadingly high read counts — this is especially a problem at a high cycle number when reagents are a limiting factor^50,56^. It is understood that variability increases with increased PCR cycles and could be mitigated by reduction, but since DNA libraries need to be large enough to be quantified (and for NGS workflow to be successful), a sufficient number of cycles must be carried out^56^. This is a significant impediment, though many labs are searching for alternate methods of DNA library quantification that come with less bias and are developing PCR-free NGS pipelines^56^. Another type of error that may lead to false positives in NGS results is introduction of erroneous sequences. This can be a result of point mutations, chimeric molecule or heteroduplex formation during PCR, or errors during sequencing^50,55,57,58^. These PCR artifacts, which may correspond to other species’ sequences, may then be amplified to the extent that they appear a positive hit for an adulterant^57^. Some of these PCR bias and bioinformatic issues have the ability to be addressed by corrective algorithms, but they must be designed for exhaustive DNA sequence libraries, complete in inclusion of all relevant species and species haplotypes^59^. Though efforts to address bias are ongoing, genetic identification is an important first step to purity screening of supplements. Quick identification tools can be used to flag samples for further quantitative analysis and assessment of potential risk. For example, trace amounts of allergens from botanical materials can elicit severe reaction in some people, thus sensitivity in detection tools is advantageous^60,61^.

LC-MS/MS is the most relevant tool to protein source identification, as amino acid sequencing allowed us to test for presence of actual protein instead of using DNA as a proxy. However, we observed very different results between the LC-MS/MS run where we first performed a TRIzol® protein isolation on protein powders compared to when we did not. Absence of protein identification other than the main protein ingredients suggested that the protein isolation acted as sieve for what could be potentially low concentration additional ingredients. Interestingly, in five cases, a main protein source that was detected in the non-isolated batch, was not detected in the TRIzol® isolated one. This may also suggest a loss of protein during isolation due to something like the poor solubility we encountered with some materials^41^. These contrasting results exemplify the multiple instances in a methodological pipeline that can dictate success in detection. Nevertheless, recent application-oriented research is demonstrating the utility and sensitivity of LC-MS/MS^62,63^. Idiosyncrasies of any given ingredient must be understood as they apply to a given analysis method, and analytical tests should be both fit for purpose and supported by reference materials to minimize false negatives.

Ultimately, orthogonality in testing of dietary supplements is the most comprehensive approach to authentication (i.e., the use of multiple ingredient identity testing tools that use different approaches to measurement). As mentioned, the protein supplement industry is plagued by “protein spiking” tactics designed to exploit the inaptitude of indirect protein measurement assays currently in use. The addition of protein source identification to protein supplement quality testing is the first step to more effective quality assurance programs.

## Conclusion

Plant-based protein powders are a rapidly growing section of the dietary supplement industry and fit for purpose authentication tools are required to address all possible risks to product quality. Molecular diagnostic tools like targeted PCR and NGS show promise in being able to identify main ingredients as well as possible adulterants, bearing in mind that certain biases may make quantitative judgements difficult. The sensitivity of these tools makes them a good starting point to screen for ingredients and use data to make decisions about further investigation. LC-MS/MS has also been demonstrated to be a useful tool in protein identification and has the added advantage of measuring the target of interest directly (actual protein from the source) as opposed to using DNA as a proxy. However, we demonstrate that other parts of the methodological pipeline such as protein isolation may influence ability to detect lower concentration adulterants.

This study was not meant to be a market survey leading to a decisive judgement of product authenticity in the industry, but rather an assessment of the applicability of certain diagnostic approaches to plant-based protein powders. We believe this is effectively achieved in the form of a case study. We understandably capture variation in processing of similar ingredients from different companies, as well as different combinations of the most popular plant-based protein ingredients. As a result of this, we can gain insight into the general utility of different authentication technologies and begin to understand the nuances of different products and ingredients that may make one approach more effective than another. With orthogonality of testing using multiple fit for purpose diagnostic tools, a comprehensive profile of any protein powder product can be constructed that will serve as an effective safeguard against sub-par quality products being sold to consumers.

## Methods

### Sample collection & organization

Thirty-three samples of commercial plant-based protein powders were purchased either from amazon.ca or store shelves (supermarket/health/drug stores), representing an array of the main sources of protein — yellow pea protein (*Pisum sativum*), brown rice protein (*Oryza sativa*), soybean protein (*Glycine max*), hemp seed protein (*Cannabis sativa*), quinoa protein (*Chenopodium quinoa*), chia seed protein (*Salvia hispanica*), and pumpkin seed protein (*Cucurbita sp*.) — in both single and multi-ingredient products, from multiple companies. About 60 g of each powder was placed into a sterile plastic bag and assigned a random sample number before conducting a blind experiment for the following reported analyses. Thirteen voucher specimens representing the main protein sources as well as potential adulterants were obtained and morphologically identified by a botanist to serve as reference samples. An additional *Bos taurus* protein reference (milk) was obtained from the Elora Research Farms Rso (University of Guelph affiliated). All references are listed in Supplementary Table S2.

### DNA extraction & quantification

DNA was extracted using the NucleoSpin® Plant II “Genomic DNA from Plant” Kit (Macherey-Nagel GmbH & Co. KG, Düren, Germany). Extractions were performed using 60 mg of dry starting material for each sample. Aside from two reagent volume amendments (i.e., 600 µL of lysis buffer and 60 µL elution buffer), protocol was followed as according to the manufacturer’s instructions. Fluorometric quantification was carried out using a Qubit™ 3.0 Fluorometer with a Qubit™ dsDNA HS assay kit, according to the manufacturer’s instructions (Life Technologies, Carlsbad, CA).

### Conventional PCR and sanger sequencing for thirteen target species

Each sample was first run on a PCR assay: single-plex reactions using each of thirteen primer sets, carried out in triplicate. Primers were designed and optimized by the Natural Health Product Research Alliance (NHPRA) (University of Guelph, Canada) for the seven main protein sources — *Pisum sativum*, *Oryza sativa*, *Glycine max*, *Cannabis sativa*, *Chenopodium quinoa*, *Salvia hispanica*, and *Cucurbita* sp. — and six other botanicals that were explored as possible adulterants to powders — *Zea mays* (maize), *Triticum aestivum* (wheat), *Vica faba* (fava bean), *Cicer arietinum* (chickpea), *Cyamopsis* sp. (guar or cluster bean) and *Medicago sativa* (alfalfa grass) (Supplementary Table S2). PCR regimes using an Eppendorf Mastercycler Pro (Eppendorf, Hamburg, Germany) were carried out using protocol: one cycle incubation (3 min at 95 °C), 35 cycles of denaturation, annealing and elongation (1 min at 95 °C, 1 min at unique temperature, 1 min at 72 °C), one cycle hold (5 min at 72 °C), and hold at 4 °C.

PCR products were used as template for a BigDye™ Terminator v3.1 Cycle Sequencing Kit (Thermo Fisher Scientific, Waltham, MA) reaction, and sent for sequencing at the Advanced Analysis Centre (AAC) Genomics facility at the University of Guelph, Canada. The BigDye™ Terminator PCR protocol was as follows: one cycle (2 min at 96 °C), 30 cycles (30 s at 96 °C, 15 s at 55 °C, 4 min at 60 °C), and hold at 4 °C. Sequencing was carried out using an Applied Biosystems 3730xl DNA Analyzer (Applied Biosystems, Foster City, CA). Sequence data was edited using the software CodonCode Aligner V3.7.1.2 (CodonCode Corporation, Centerville, MA). Consensus sequences were aligned with known herbarium vouchers and reference material sequences curated at the NHPRA, University of Guelph. Statistical phylogenetic analysis was completed using Geneious software (Biomatters Ltd, Auckland, New Zealand).

### Hydrolysis probe based real time PCR for GMO soy and whey protein

A hydrolysis probe based test for GMO-soy was carried out for all samples on a LightCycler® 480 Instrument II (Roche Diagnostics, Rotkreuz, Switzerland), investigating the presence of GMO soy. The probe targeted the CP4 gene (glyphosate tolerance in GMO soy) (primers and probe available from NHPRA, University of Guelph — Supplementary Table S2). Furthermore, another probe-based screen was run on all samples investigating the presence of whey; the probe targeted a sequence unique to *Bos taurus* (primers and probe available from NHPRA, University of Guelph — Supplementary Table S2). Each reaction was run in quadruplicate. All samples were screened using the two probes to investigate plant-only claims of products and level of GMO presence (products either claimed non-GMO or did not note GMO inclusion).

### Next generation sequencing

Next Generation Sequencing (NGS) was carried out by targeted amplicon sequencing with universal plant *ITS* primers on an Illumina Miseq Desktop Sequencer (Illumina, San Diego, CA) at AAC Genomics, University of Guelph. In the case of unsuccessful identification of main protein ingredients, NGS was carried out using universal *rbcL* primers (primers available from NHPRA, University of Guelph — Supplementary Table S2). NGS workflow was based on the 16S Metagenomic Sequencing Library Preparation protocol from Illumina^64^, with the amendment of primer selection. Workflow is summarized as follows: 1. Extract, purify and normalize DNA from samples; 2. Amplify DNA target region using universal primers and KAPA HiFi HotStart ReadyMix (25 cycles) (Kapa Biosystems, Wilmington, MA); 3. Carry out PCR cleanup with AMPure XP beads to size select the correct amplicon by removing excess primers and nucleotides; 4. Run index PCR to attach dual indices and Illumina sequencing adapters (8 cycles); 5. Carry out second PCR cleanup with AMPure XP beads (Beckman Coulter, Brea, CA); 6. Quantify each sample library using Qubit™ 3.0 Fluorometer and normalize library concentration to 4 nM with ddH_2_0 and pool the samples attached with dual indices; 7. Carry out sequencing on Illumina Miseq Desktop Sequencer; 8. Use CLC Genomics Workbench (Qiagen, Hilden, Germany) for data analysis to get the individual sequences of Operational Taxonomic Units (OTU) with the quantitative metrics of read count. The resulting sequences were manually searched on GenBank to get taxonomic classifications of individual sequences.

### Protein isolation and digestion, and LC-MS/MS amino acid sequencing and analysis

Two LC-MS/MS runs were carried out: one completing the trypsin digestion on protein powder without a prior protein isolation, and one preceded by a protein isolation. We isolated proteins using the TRIzol® reagent according to the user guide.

All protocols involved in the LC-MS/MS sample preparation and analysis were provided by the AAC Mass Spectrometry Facilities, University of Guelph. The trypsin digestion protocol was completed as follows: 10 mg of sample was dissolved in 1 mL of 50 mM sodium bicarbonate. Next, 100 µL of that dissolved sample was resuspended in 900 µL of 50 mM sodium bicarbonate. Then, 15 µL of dithiothreitol (DTT) was added to 150 µL of that sample and incubated for one hour at 60 °C. Samples were digested with lysine C for three hours, followed by a 20 µL addition of trypsin (Princeton Separations Adelphia, NJ) and incubation overnight at 37 °C. Digestion was stopped via addition of trifluoroacetic acid, and solutions were dried under vacuum.

Protein identification was carried out for each sample via amino acid sequencing on an Agilent 1200 HPLC liquid chromatograph interfaced with an Agilent UHD 6530 Q-Tof mass spectrometer (Agilent Technologies, Santa Clara, CA) (at AAC Mass Spectrometry Facilities, University of Guelph). A C18 column (Agilent Advance Bio Peptide Map, 100 mm × 2.1 mm 2.7 µm) was used for chromatographic separation with solvents A and B (Solvent A: dH_2_O with 0.1% formic acid; Solvent B: acetonitrile with 0.1% formic acid). Procedure for the mobile phase gradient included: initial conditions, 2% B, increasing to 45% B in 40 min and then to 55% B in next 10 min, followed by column wash at 95% B and 10 min re-equilibration. The first two and last five minutes of the gradient were sent to waste (not the spectrometer). A 0.2 mL/min flow rate was maintained. Mass spectrometer electrospray capillary voltage was maintained at 4.0 kV and the drying gas temperature at 350 °C with a flow rate of 13 L/min. Nebulizer pressure was 40 psi and the fragmentor was set to 150. Nitrogen was used as nebulizing, drying gas, and collision-induced dissociation gas. Mass-to-charge ratio was scanned across the m/z range of 300–2000 m/z in 4 GHz (extended dynamic range positive-ion auto MS/MS mode). In each cycle, three precursor ions were selected for fragmentation. Externally calibration of the instrument was carried out with the ESI TuneMix (Agilent, Mississauga, ON). The sample injection volume was 100 µL.

Gathered raw data of amino acid sequences was loaded into PEAKS 8 software (Bioinformatic Solutions Inc., Waterloo, Canada). Data refinement was carried out in two steps. Amino acid sequence data was subjected to deNovo sequencing, as well as database searching of Swiss-Prot (database searching using a 1% FDR). Methionine oxidation and carbamidomethylation of cysteine residues were considered within the search parameters. Other instrument parameters were set as follows in PEAKS. Precursor Mass Error Tolerance (for parent ions): 15ppm, Fragment Mass Error Tolerance (for fragment ions): 0.05 Da, Digest: Trypsin, Oxidation: 15.99, Deamination: 0.98, Variable Post Translational Modification Per Peptide: 3, Report Number of Peptides: 5, Data Refine Dependencies: 1. An ingredient identification was included in this first set of data if there were at least two protein hits for a given species. From this initial compiled list of proteins, a second manual curation was carried out where each protein was researched and only retained as a positive ingredient identification if the protein was unique to that species. A protein that was found to be ubiquitous among multiple species (i.e., matched to multiple species entries in Swiss-Prot) was eliminated; thus, only unique, diagnostic proteins were retained.

## Supplementary information

Supplementary Table S1 and S2
