# Supplementary material for: Investigating appropriate molecular and chemical methods for ingredient identity testing of plant-based protein powder dietary supplements
Source: Sci Rep. 2019 Aug 20;9:12130. doi: 10.1038/s41598-019-48467-9 (PMC6702227; doi:10.1038/s41598-019-48467-9)
Supplement: Supplementary file 1 — Supplementary Table S1 and S2 [file 41598_2019_48467_MOESM1_ESM.pdf]

Investigating appropriate molecular and chemical methods for ingredient identity testing of plant-based protein powder dietary supplements

Adam C Faller[1*], Thirugnanasambandam Arunachalam[1+], Dhivya Shanmughanandhan[1+], Prasad Kesanakurti[1+], Hanan R Shehata[1+], Subramanyam Ragupathy[1], Steven G Newmaster[1]

*afaller@uoguelph.ca
[+]these authors contributed equally to this work

Supplementary Information Attached (Two Tables)

Supplementary Table S1. Individual adulterant detection results for PCR with Sanger Sequencing, NGS and LC-MS/MS.

	PCR + SEQ	NGS				LC-MS/MS
**SAMPLE**	**Targeted amplification results (adulterant)**	**Region**	**# of reads used for building OTU clusters**	**Major species identified**	**Other plant species (less than 1% reads)**	**Amino acid sequencing results (adulterant) (species in brackets indicate a peptide with multiple hits)**
**P1**	*Pisum sativum*, *Cucurbita* sp.	*ITS*	192577	*Glycine max* (99.8% reads)	*Phaseolus vulgaris; Pisum sativum; Cannabis sativa; Salvia* sp.	*Atriplex nummularia, Pseudomonas aeruginosa, (Canavalia ensiformis / Canavalia gladiata), (Daucus carota / Oryza sativa), Pisum sativum, Paracoccidioides lutzii / Schizosaccharomyces pombe*
**P2**		*ITS*	234474	*Glycine max* (99.7% reads)	*Phaseolus vulgaris; Cannabis sativa; Xanthium* sp.; *Salvia* sp.;	*(Canavalia ensiformis / Canavalia gladiata)*
**P3**		*ITS*	139119	*Salvia* sp. (99.7% reads)	*Malvastrum* sp.; *Brassica* sp.; *Amaranthus* sp.; *Sida* sp.; *Nicandra physalodes; Conyza sumatrensis; Boerhavia* sp.	*Glycine max*
**P4**	*Glycine max*	*ITS*	161524	*Cucurbita* sp. (99.5% reads)	*Cannabis sativa; Secale cereale; Glycine max; Pisum sativum; Helianthus* sp.; *Hordeum vulgare; Triticum aestivum; Vaccinium* sp.	*Glycine max*
**P5**		*ITS*	177328	*Cannabis sativa* (99.9% reads)	*Pisum sativum; Lavandula angustifolia; Salvia* sp.	
**P6**		*ITS*	236001	*Cannabis sativa* (99.9% reads)	*Pisum sativum; Salvia* sp.; *Medicago* sp.	
**P7**	*Triticum* sp.	*ITS*	117891	*Cannabis sativa* (98.8% reads)	*Brassica* sp.; *Linum usitatissimum; Borago officinalis; Phaseolus vulgaris; Malva* sp; *Helianthus* sp.; *Amaranthus* sp.; *Pisum sativum; Erodium* sp.; *Sonchus* sp.; *Fragopyrum esculentum;Bassia scoparia*	
**P8**	*Chenopodium* sp.	*ITS*	112721	*Cannabis sativa* (99.9% reads)	*Pisum sativum; Sonchu* sp.; *Salvia* sp.;	*Pisum sativum / Arabidopsis thaliana / Atriplex nummularia / Oryza sativa* subsp.
**P9**	*Cucurbita* sp.	*ITS*	163450	*Cannabis sativa* (99.9% reads)	*Pisum sativum; Sinapis alba; Medicago sativa; Lotus* sp.;	*(Pisum sativum / Arabidopsis thaliana), (Glycine max / Pisum sativum / Oryza sativa)*
**P10**	*Glycine max*	*ITS*	143609	*Cannabis sativa* (99.9% reads)	*Linum usitatissimum; Pisum sativum; Borago officinalis; Vicia* sp.; *Lathyrus* sp.; *Brassica* sp.; *Lens* sp.; *Raphanus sativus; Coriandrum sativum*	
**P11**		*ITS*	154966	*Cucurbita* sp. (99.3% reads)	*Cannabis sativa; Pisum sativum; Cyamopsis* sp.; *Salvia* sp.;	
**P12**	*Cyamopsis* sp.	*ITS*	184765	*Cucurbita* sp. (99.2% reads)	*Pisum sativum; Citrullus* sp.; *Glycine max;*	*Glycine max / Daucus carota*
**P13**	*Cucurbita* sp.	*rbcL*	8653	*Oryza sativa* (99.8% reads)	*Echinochloa* sp.	*Cucurbita maxima, Arabidopsis thaliana, Mus musculus*
**P14**		*rbcL*	6195	*Oryza sativa* (99.8% reads)	*Echinochloa* sp., *Indigofera tinctoria*	*Zea mays, Arabidopsis thaliana*
**P15**	*Cyamopsis* sp.	*rbcL*	104114	*Oryza sativa* (99.8% reads)	*Echinochloa* sp.	
**P16**	*Cucurbita* sp., *Chenopodium* sp.	*rbcL*	608	*Oryza sativa* (100% reads)		*Hordeum vulgare, Zea mays*
**P17**	*Pisum sativum*, *Cucurbita* sp.	*rbcL*	106421	*Oryza sativa* (99.8% reads)	*Echinochloa* sp., *Euphorbia* sp.,	*Zea mays, Atriplex nummularia, Pisum sativum*
**P18**	*Pisum sativum*	*rbcL*	95899	*Oryza sativa* (99.6% reads)	*Echinochloa* sp.,	*Zea mays*
**P19**		*rbcL*	8305	*Oryza sativa* (99.7% reads)		
**P20**		*ITS*	169077	*Pisum sativum* (99.9% reads)	*Citrus* sp.; *Brassica* sp.	*Vicia faba*
**P21**		*ITS*	164473	*Pisum sativum* (99.9% reads)	*Cirsium arvense*	*Cicer arietinum, Arabidopsis thaliana, Glycine max*
**P22**		*ITS*	207613	*Pisum sativum* (99.9% reads)		*Vicia faba, Cicer arietinum, Arabidopsis thaliana, Glycine max, Medicago sativa*
**P23**		*ITS*	215580	*Pisum sativum* (99.9% reads)		*Vicia faba, Cicer arietinum, Arabidopsis thaliana*
**P24**	*Triticum* sp.	*ITS*	144217	*Pisum sativum* (99.9% reads)	*Vicia* sp.; *Glycine* sp.	*Vicia faba, Glycine max*
**P25**		*ITS*	233779	*Pisum sativum* (99.9% reads)	*Glycine* sp.	*Vicia faba, Myrmica ruginodi, Arabidopsis thaliana*
**P26**		*ITS*	206237	*Pisum sativum*(67.2% - 138414reads); *Cannabis sativa* (26.2% - 53984 reads); *Salvia* sp. (6.4% - 13180 reads);	*Linum usitatissimum; Cyamopsis* sp.; *Taraxacum* sp.; *Vaccinium* sp.; *Beta vulgaris; Malva pusilla;*	*Vicia faba, Arabidopsis thaliana, Petroselinum crispum*
**P27**		*ITS*	253886	*Pisum sativum* (99.8% reads)	*Linum usitatissimum; Spinacia* sp.; *Brassica oleraceae; Vaccinium* sp.; *Cicer arietinum; Cyamopsis* sp.	*Vicia faba, Cicer arietinum, Arabidopsis thaliana*
**P28**		*ITS*	Unsuccessful Reaction			
**P29**		*ITS*	297917	*Pisum sativum* (99.9% reads)	*Moringa* sp.; *Brassica* sp.; *Medicago* sp.	*Vicia faba, Cicer arietinum, Arabidopsis thaliana*
**P30**		*ITS*	241455	*Pisum sativum* (98% reads); *Medicago sativa* (1.6% reads);	*Salvia* sp.; *Brassica* sp.; *Capsilla bursa-pastoris; Descurainia sophia; Rubus* sp.; *Rosa* sp.; *Spinacia* sp.; *Tripleurospermum; Cotesia* sp.; *Lamium* sp.; *Amaranthus* sp.	*Arabidopsis thaliana*
**P31**	*Triticum* sp.	*ITS*	122966	*Pisum sativum* (99% reads); *Medicago* (0.9% reads)	*Medicago* sp.; *Brassica* sp.; *Plukenetia* sp.; *Spinacea* sp.; *Taraxacum* sp.; *Cicer arietinum; Cannabis* sp.; *Stellaria* sp.; *Lens* sp.	*Cicer arietinum, Arabidopsis thaliana*
**P32**		*ITS*	133003	*Pisum sativum* (99.6% reads)	*Cannabis sativa*	*Vicia faba*
**P33**	*Pisum sativum*	*ITS*	Unsuccessful Reaction			*Pisum sativum, (Canavalia ensiformis / Canavalia gladiata), Oryza sativa*

Supplementary Table S2. List of reference samples and associated primer design.

Testing Technique	Reference Sample	Primer sequence	Primer Design Region	Amplicon Length (bp)	Sample notes
Targeted PCR, NGS, LC-MS/MS	*Pisum sativum*	F- 5' GCGTTCTCTTCTGTGCCAAA 3'<br>R- 5' TATCCGTTGCCGAGAGTCAT 3'	*ITS*	152	From reference library curated at University of Guelph
	Oryza sativa	F- 5' CGTCAAGGAACACAGCGATAC 3'<br>R- 5' GATTCTGCAATTCACACCAGGTA 3'	*ITS*	171	
	Glycine max	F- 5' GCAGAATCCCGTGAACCATC 3'<br>R- 5' GAACCCAGATTTCAACCAACCA 3'	*ITS*	184	
	Cannabis sativa	F- 5' GTATGGTCTCAAGCAGGAAGTATT 3'<br>R- 5' TTAGCCAACGATCCAATCAGAG 3'	*matK*	200	
	Chenopodium quinoa	F- 5' CGAGTCTTTGAACGCAAGTTG 3'<br>R- 5' CCTTGTCTACCACCTATTGC 3'	*ITS*	200	
	Salvia hispanica	F- 5' AACCAAACGAAGCATCCTCCC 3'<br>R- 5' GCAATTCACACCAAGTATCGCATT 3'	*ITS*	153	
	Cucurbita sp.	F- 5' CGACCCGTGAACGTGTTTAC 3'<br>R- 5' TGCCGAGAGTCGTTGTGAAT 3'	*ITS*	185	
	Zea mays	F- 5' CTTCCATTGTGGGTAACGTATTTG 3'<br>R- 5' AAGGACGACCGTACTTGTTCA 3'	*rbcL*	153	
	Triticum aestivum	F- 5' CGAAGGCGTCAAGGAACA 3'<br>R- 5' ATTCACACCAGGTATCGCA 3'	*ITS*	160	
	Vicia faba	F- 5' TGGCAGCATTCCGAGTAACT 3'<br>R- 5' TTCTCCAGGAACAGGCTCTATC 3'	*rbcL*	170	
	Cicer arietinum	F- 5' TTCTTCAAGGATCCTCTCATTC 3'<br>R- 5' TGAATGTTGGGATAATTGGTTT 3'	*matK*	180	
	Cyamopsis sp.	F- 5' CCTTTGGTTGGGAGTTGTCTG 3'<br>R- 5' CGATGCGAGAGCCGAGATAT 3'	*ITS*	191	
	Medicago sativa	F- 5' GTTCAGAGGAAGACGACATAGTG 3'<br>R- 5' GTTGCCGAGAGTCATTCTATATCA 3'	*ITS*	168	
NGS	All reference samples above	F-5'TCGTCGGCAGCGTCAGATGTGTATAAGAGACAGATGCGATACTTGGTGTGAAT 3'<br>R-5'GTCTCGTGGGCTCGGAGATGTGTATAAGAGACAGTCCTCCGCTTATTGATATGC 3'	*ITS*	400	
		F- 5'TCGTCGGCAGCGTCAGATGTGTATAAGAGACAGCGAGGGAATTAGGGGTTCCT 3'<br>F-5'GTCTCGTGGGCTCGGAGATGTGTATAAGAGACAGTTGCTCGGTGAATGTGAAGA 3'	*rbcL*	120	
Hydrolysis Probe QPCR, LC-MS/MS	*Bos taurus*	F- 5' GATAACAGCGCAATCCTATTCAAG 3'<br>F- 5' GAACTCAGATCACGTAGGACTTT 3'<br>P- 5' /56-FAM/TGATCCAAC /ZEN/ATCGAGGTC GTAAACCC/3IABkFQ/ 3'	QPCR: *16S* region, 140 bp amplicon		Milk sample obtained from Dairy Cows #4316 and #4634 (pooled sample) from the Elora Research Farms Rso (U of G Affiliated)
Hydrolysis Probe QPCR	*Glycine max* (GMO)	F- 5' TCCCGCTCTAGCGCTTCAAT 3'<br>R- 5' TCGAGCAGGACCTGCAGAA 3'<br>P- 5' CTGAAGGCGGGAAACGACAATCTG 3'	QPCR: *CP4* region, 139 bp amplicon		From reference library curated at University of Guelph
